# Actinomycetes from Red Sea Sponges: Sources for Chemical and Phylogenetic Diversity

**DOI:** 10.3390/md12052771

**Published:** 2014-05-12

**Authors:** Usama Ramadan Abdelmohsen, Chen Yang, Hannes Horn, Dina Hajjar, Timothy Ravasi, Ute Hentschel

**Affiliations:** 1 Department of Botany II, Julius-von-Sachs Institute for Biological Sciences, University of Würzburg, Julius-von-Sachs-Platz 3, Würzburg D-97082, Germany; E-Mail: hannes.horn@uni-wuerzburg.de; 2 Division of Chemical & Life Sciences and Engineering and Division of Applied Mathematics and Computer Science, King Abdullah University of Science and Technology, Thuwal 23955-6900, Saudi Arabia; E-Mails: chen.yang@kaust.edu.sa (C.Y.); dina.hajjar@kaust.edu.sa (D.H.); timothy.ravasi@kaust.edu.sa (T.R.)

**Keywords:** PKS I, PKS II, NRPS, Red Sea, sponges, actinomycetes, bioactivity

## Abstract

The diversity of actinomycetes associated with marine sponges collected off Fsar Reef (Saudi Arabia) was investigated in the present study. Forty-seven actinomycetes were cultivated and phylogenetically identified based on 16S rRNA gene sequencing and were assigned to 10 different actinomycete genera. Eight putatively novel species belonging to genera *Kocuria*, *Mycobacterium*, *Nocardia*, and *Rhodococcus* were identified based on sequence similarity values below 98.2% to other 16S rRNA gene sequences available in the NCBI database. PCR-based screening for biosynthetic genes including type I and type II polyketide synthases (PKS-I, PKS-II) as well as nonribosomal peptide synthetases (NRPS) showed that 20 actinomycete isolates encoded each at least one type of biosynthetic gene. The organic extracts of nine isolates displayed bioactivity against at least one of the test pathogens, which were Gram-positive and Gram-negative bacteria, fungi, human parasites, as well as in a West Nile Virus protease enzymatic assay. These results emphasize that marine sponges are a prolific resource for novel bioactive actinomycetes with potential for drug discovery.

## 1. Introduction

Sponges (*Porifera*) are the oldest, evolutionarily ancient multicellular phylum with a fossil record dating back to Precambrian times [1]. The phylum *Porifera* consists of three major classes, Hexactinellida (glass sponges), Calcarea (calcareous sponges) and Demospongiae (demosponges), with the last group representing 85% of all living sponges [2]. Sponges populate tropical reefs in great abundance but also the polar latitudes as well as fresh water lakes and rivers [3,4]. Sponges have developed intimate contact with diverse microorganisms such as viruses, bacteria, archaea, fungi, protozoa, and single-celled algae and the nature of the sponge-microbe interaction is manifold [5]. The microbial distribution in most sponges follows a general pattern with the photosynthetically active microorganisms such as *Cyanobacteria* located in the outer light exposed layers while heterotrophic and possibly autotrophic bacteria inhabiting the inner core [6]. So far, at least 32 bacterial phyla and candidate phyla were described from marine sponges by both cultivation-dependent and cultivation-independent techniques; with the most common phyla being *Acidobacteria*, *Actinobacteria*, *Chloroflexi*, *Cyanobacteria*, *Gemmatimonadetes*, *Nitrospira*, Planctomycetes, *Proteobacteria*, (*Alpha*, *Delta*, *Gamma* subclasses) and Spirochaetes [1,3].

**Figure 1:**
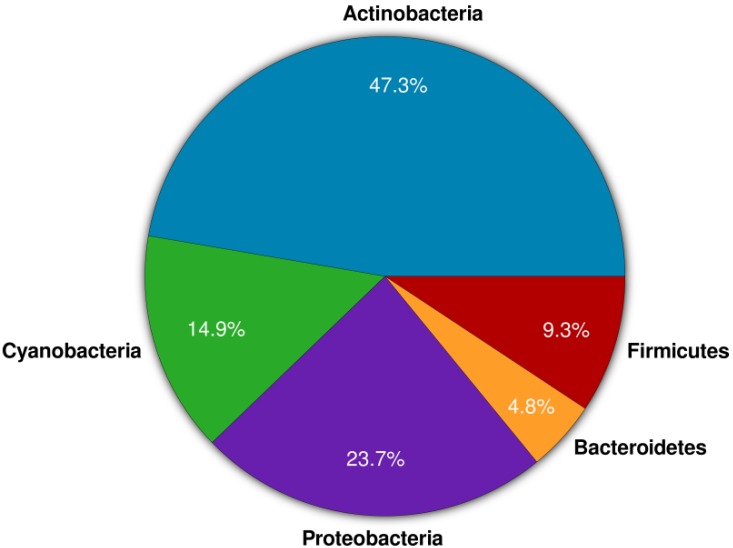
Percentage distribution of compounds produced by sponge-associated bacteria (data collected from MarinLit 2013 and literature).

The phylum *Actinobacteria* represents one of the largest taxonomic units among the 18 major lineages currently recognized within the domain bacteria [7]. The subclass Actinobacteridae includes the order Actinomycetales, members of which are commonly referred to as actinomycetes. These are Gram positive bacteria characterized by their ability to form branching hyphae at some stages of their development [8]. Within the order Actinomycetales, approximately 49 families have been recognized with the most common ones being *Actinomycetaceae*, *Actinoplanaceae*, *Dermatophilaceae*, *Frankiaceae*, *Mycobacteriaceae*, *Micromonosporaceae*, *Nocardiaceae*, and *Streptomycetaceae*, comprising altogether 147 genera [9,10]. Actinobacteria are widespread in nature and have been recovered from a wide variety of terrestrial habitats, where they exist as saprophytes, symbionts or pathogens [11,12,13]. Actinomycetes have been cultivated from the marine environment including sea water [14], marine snow, and marine sediments [15]. Actinomycetes have also been cultivated from different marine invertebrates [16,17,18], with the majority being isolated from sponges [19,20]. Marine actinomycetes produced a multitude of novel lead compounds with medicinal and pharmaceutical applications. Figure 1 shows the percentage distribution of compounds obtained from marine sponge-associated bacteria. Here, actinomycetes account for approximately half of the natural products (MarinLit database 2013 (John Blunt, MarinLit, University of Canterbury, New Zealand) [21,22]), (Figure 1). Biological activities such as antibacterial, antifungal, antiparasitic, antimalarial, immunomodulatory, anti-inflammatory, antioxidant, and anticancer activities were reported from sponge-associated actinomycetes [23,24,25,26]. These bioactivities are represented by diverse leads of secondary metabolites including polyketides, alkaloids, peptides, and terpenes [24,25,27,28,29,30].

Polyketide synthases (PKS) and nonribosomal peptide synthetases (NRPS) are multi-domain megasynthases that are involved in the biosynthesis of a large fraction of diverse microbial natural products known as polyketides and nonribosomal peptides, respectively [31]. These enzymes are widely distributed among the actinomycetes, cyanobacteria, myxobacteria, and fungi [32]. Structurally, both PKS and NRPS are complex polypeptides organized in a modular fashion for assembling carboxylic acid and amino acid building blocks into their final products [33]. Each PKS module encodes three basic domains including ketosynthase (KS), acyltransferase (AT), and acyl carrier protein (ACP), which are involved in the selection and condensation of the correct extender unit. Similarly, NRPS modules consist of condensation, adenylation, and thiolation domains for recognition and condensation of the starter substrate [31]. In this study, actinomycetes were cultivated from marine sponge species collected from the Red Sea. The obtained actinomycetes were phylogenetically characterized based on 16S rRNA gene sequencing and their genomic potential for natural products biosynthesis as well as their biological activities in an infection context are reported.

## 2. Results and Discussion

The Red Sea is one of the most biodiverse marine environments worldwide. It is characterized by high temperature (about 24 °C in spring and up to 35 °C in summer) and high salinity (*ca.* 40.0 psu), rendering this environment physically and chemically different from most other marine ecosystems [34]. About 240 demosponges have been formally recorded from the Red Sea so far and still many more await discovery [35]. Ngugi *et al.* reported a high bacterial diversity in the Red Sea in comparison to other tropical seas [36]. However, few studies have been carried out so far to investigate actinobacterial communities from Red Sea sponges. One such example is the study by Radwan *et al.* [37] who explored the microbial diversity of two Red Sea sponges, *Hyrtios erectus* and *Amphimedon* sp., using cultivation and cultivation-independent analyses.

### 2.1. Actinomycete Isolation and Phylogenetic Identification

Twenty-five isolates were selected out of cultivated 47 isolates based on their characteristic colony morphology. The 16S rRNA genes as taxonomic markers were sequenced and sequences were blasted against the NCBI GenBank database. The results showed that the isolates belonged to 10 different genera representing six families (*Dietziaceae*, *Micrococcaceae*, *Micromonosporaceae*, *Mycobacteriaceae*, *Nocardiaceae*, *Pseudonocardiaceae*) and four suborders (Corynebacterineae, Micrococcineae, Micromonosporineae, Pseudonocardineae). Eight putatively new species were identified based on sequence similarities <98.2%, which belonged to the genera *Kocuria*, *Mycobacterium*, *Nocardia* and *Rhodococcus* (Table 1). From a taxonomic perspective, sequence similarities after BlAST analysis against type strains may even be lower. As one example, the isolate SA8 showed 96% sequence similarity to the closest type strain (*Rhodococcus trifolii*^T^). However, as type strains are not available for all obtained isolates, this taxonomically meaningful comparison remains restricted.

**Table 1:** 16S rRNA gene taxonomic affiliation of cultivated strains.

Isolate Code	Isolation Medium	Sponge Source	Sequence Length (bp)	Closest Relative by BLAST	% Sequence Similarity
SA1	M1	*Amphimedon ochracea*	1379	*Micrococcus* sp. PN13 (KF554087)	99.4
SA2	ISP2	*Amphimedon* aff. *chloros*	1435	*Micrococcus luteus* strain DAG I (KC470045)	99.7
SA3	ISP2	*Amphimedon* aff. *chloros*	1360	*Micrococcus* sp. X2Bc2 (KF465977)	99.5
SA4	ISP2	*Hyrtios erectus*	1423	*Micromonospora* sp. S6 (HF674982)	99.7
SA5	OLIGO	*Chalinula* sp.	1395	*Salinispora arenicola* CNS-205 (NR_074612)	99.9
SA6	OLIGO	*Chalinula* sp.	1372	*Salinispora arenicola* strain SCSIOZ-SH19 (KC747487)	100.0
* SA7	ISP2	*Chalinula* sp.	1338	*Nocardia* sp. W9912 (GU992878)	98.2
* SA8	ISP2	*Chalinula* sp.	1461	*Rhodococcus* equi (AB738794)	97.4
* SA9	ISP2	*Monanchora* sp.	1378	*Rhodococcus* sp. L-15 (JN006270)	97.9
SA10	M1	*Chalinula* sp.	1397	*Micrococcus* sp. PA-E028 (FJ233852)	99.8
* SA11	M1	*Monanchora* sp.	1416	*Mycobacterium* sp. CNJ879 PL04 (DQ448780)	97.6
* SA12	ISP2	*Chalinula* sp.	1374	*Rhodococcus* sp. HL-3 (JF734314)	97.9
* SA13	M1	*Monanchora* sp.	1419	*Mycobacterium* sp. CNJ879 PL04 (DQ448780)	97.8
* SA14	M1	*Amphimedon ochracea*	1341	*Kocuria* sp. PN5 (KF554079)	97.5
* SA15	M1	*Amphimedon* aff. *chloros*	1347	*Kocuria* sp. SS263-23 (JX429815)	97.3
SA16	M1	*Amphimedon* aff. *chloros*	1395	*Rothia terrae* strain F77052 (HQ908743)	99.7
SA17	ISP2	*Crella cyathophora*	1401	*Micrococcus* sp. X-48(JX997909)	99.9
SA18	M1	*Amphimedon* aff. *chloros*	1413	*Dietzia maris* strain KMGL1309-AS3 (KF740541)	100.0
SA19	OLIGO	*Chalinula* sp.	1325	*Salinispora pacifica* strain AMS365 (HQ873949)	99.9
SA20	M1	*Subera* sp.	1330	*Salinispora arenicola* strain SCSIOZ-SH19 (KC747487)	100.0
SA21	ISP2	*Subera* sp.	1443	*Saccharomonospora azurea* strain RR1 (KC855265)	99.2
SA22	OLIGO	*Subera* sp.	1337	*Salinispora pacifica* strain S34 (JX007964)	99.7
SA23	ISP2	*Amphimedon* aff. *chloros*	1396	*Saccharomonospora* sp. G2Z21 (JF806667)	99.8
SA24	M1	*Dactylospongia* aff. *elegans*	1494	*Kocuria palustris* strain LJ27 (KF515677)	99.1
SA25	OLIGO	*Hyrtios erectus*	1453	*Salinispora arenicola* strain SCSIOZ-SH11 (KC747479)	99.9

* Putatively new species.

The recoverability of actinomycetes varied between different sponge sources; for example, *Amphimedon* aff. *chloros* yielded 17 actinomycetes (six genera), while *A. ochracea* yielded only four isolates from three different genera. These numbers compare to the recovery of four actinomycetes (two genera) from *Amphimedon* sp. from Ras Mohamed (Egypt) [17], 16 actinomycete (four genera) from *Amphimedon* sp. collected from Hurghada (Egypt) [37], and zero actinomycetes from *A. complanata* collected from Puerto Rico [38]. Similarly, while Radwan *et al.* [37] isolated 18 actinomycetes (four genera) from *Hyrtios erectos*, we obtained only three actinomycetes from this sponge species, albeit collected at a different location. Contrary to previous reports [39], we did not succeed in isolating actinomycetes from *Xestospongia* aff. *testudinaria*. This sporadic isolation of actinomycetes could be explained by environmental factors that would influence the diversity, abundance and recoverability of actinomycetes from sponges. The observed differences also highlight the importance of using a wide range of media to increase the isolation efficiency of marine sponge-associated actinomycetes.

**Figure 2:**
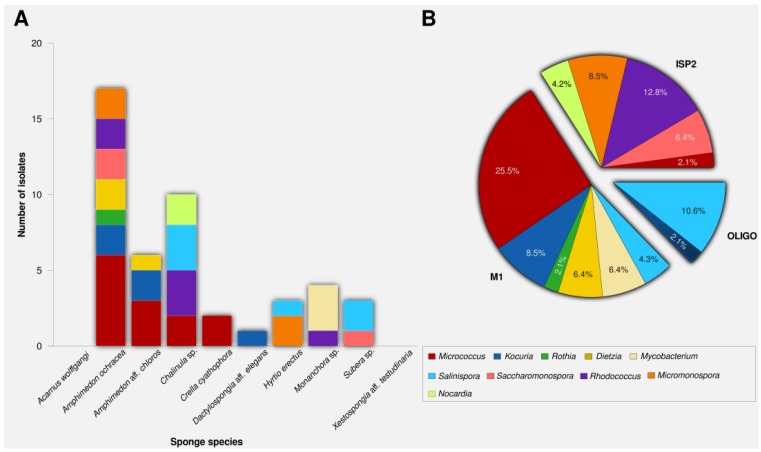
Number of actinomycete isolates (**A**) per sponge species, (**B**) per isolation media.

M1, ISP2 and OLIGO media were chosen for actinomycete cultivation based on previous experience and literature reports [17,40]. M1 medium produced the highest number of actinobacterial colonies (25), followed by ISP2 (16), while only six isolates were recovered on OLIGO (Figure 2B). Zhang *et al.* demonstrated that the lack of free amino acids resulted in low recovery of marine actinobacteria [19]. Accordingly, peptone was added to M1 medium which resulted in both, a higher number of actinomycetes and recovery of more genera. Consistent with previous studies [38,41], the genera *Rhodococcus*, *Micromonospora*, and *Nocardia* were cultivated preferentially on ISP2, while *Salinispora* grew better on oligotrophic media [42,43].

The genus *Micrococcus* was represented by the highest number of isolates (14) which is likely due to their fast growing nature, rendering them easy to isolate. The second most abundant genus (7) was *Salinispora* which is frequently isolated from sea water, sediments, as well as sponges [43,44]. The other genera belonged to *Rhodococcus* (6), *Kocuria* (5), *Micromonospora* (4), *Dietzia* (3), *Saccharomonospora* (3), *Mycobacterium* (2), *Nocardia* (2), and *Rothia* (1).

**Figure 3:**
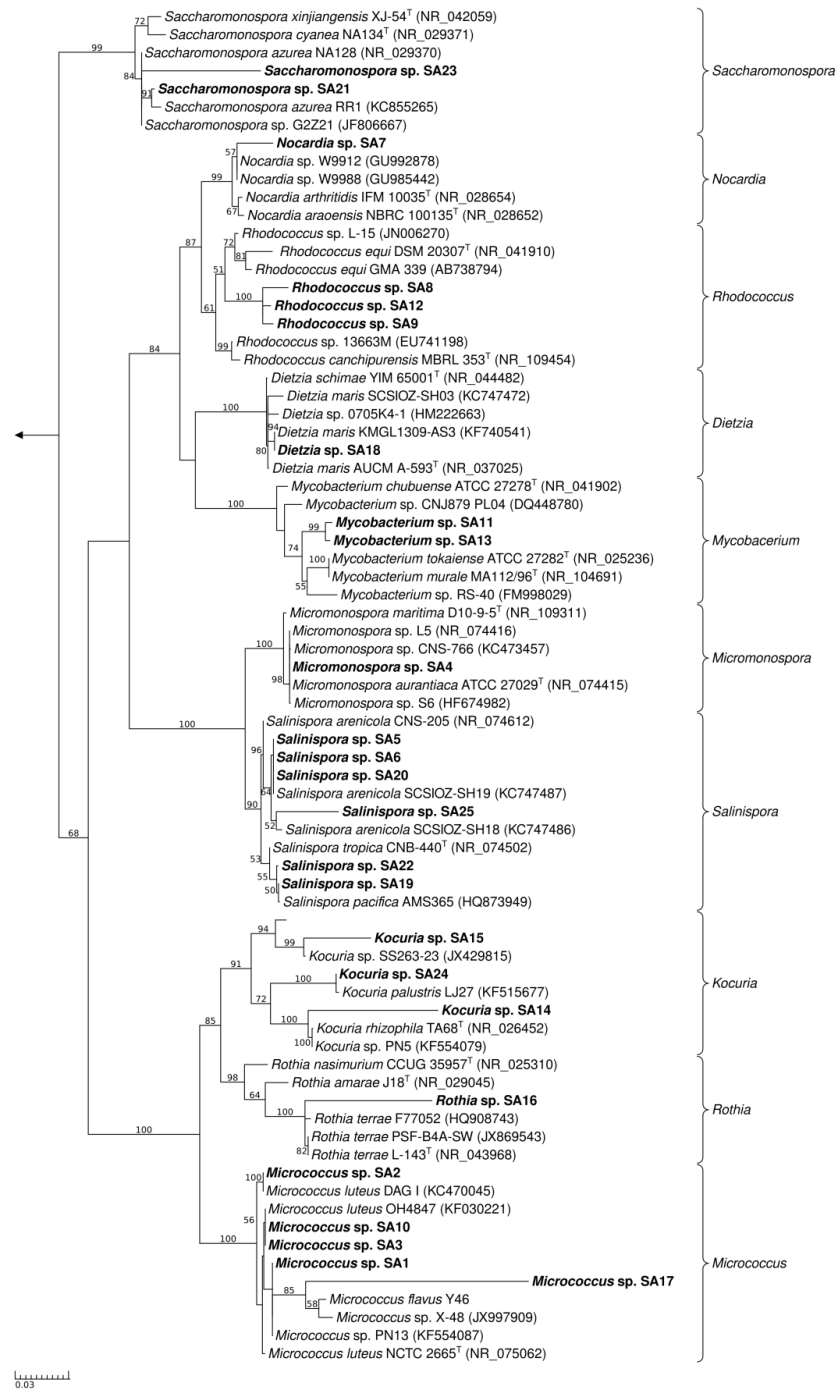
Maximum-likelihood tree of the actinomycete isolates SA1-SA25 (in bold) and their closest phylogenetic relatives based on the 16S rRNA gene marker. Brackets indicate genus-level assignment. Bootstrap values (1000 resamples) are given in percent at the nodes of the tree (greater than 50). The arrow points to the outgroup (*Escherichia coli* KTCT 2441^T^).

A maximum-likelihood tree was calculated for the 25 isolates with the nearest sequence relatives from a Blast run included (Figure 3). The *Rhodococcus* isolates sp. SA8, SA9 and SA12 formed a distinct cluster. The high similarity and high bootstrap value (100) along with a multifurcation in the tree suggests that the isolates represent the same species. The isolates SA11, SA13, SA14, and SA15 form distinct clades in the genera *Mycobacterium* and *Kocuria*. Interestingly, isolate SA7 falls within the genus *Nocardia* and also shows the lowest level of similarity (98.2%). In this case, further phenotypic and genotypic characterization may be needed to validate the exact taxonomic position of this isolate which might be a novel species within the genus *Nocardia*.

### 2.2. PCR-Screening for PKS and NRPS Domains

Twenty-five actinomycetes were tested using degenerate PCR primers for the presence of polyketide synthases Type I and II (PKS-I and PKS-II) and nonribosomal peptide synthetases (NRPS). At least one type of biosynthetic gene sequence was recovered from 20 actinomycete isolates (80%), (Table 2). All three types of biosynthetic genes were found in the actinomycetes (7) belonging to genera *Micromonospora* and *Salinispora*. This is unsurprising since *Micromonospora* and *Salinispora* are well known for their natural product diversity encompassing different metabolite classes [25,27,45]. NRPS biosynthetic genes were identified in 19 isolates (76%), while PKS-I genes were detected in 12 strains (48%), and PKS-II genes in eight strains (32%). NRPS and PKS sequence diversity have been reported in marine actinomycetes isolated from different marine environments including marine caves, marine sediments, and marine sponges, where these sequences were detected in up to 90% of the tested strains [46,47].

**Table 2:** NRPS and PKS results of cultivated strains.

Isolate Code	Closest Relative by BLASTX (Sequence Length bp, % Sequence Similarity)		
	NRPS	PKS I	PKS II
SA1	Amino acid adenylation domain-containing protein [*Micrococcus luteus* (CP001628)] (347, 51)	-	-
SA2	Non-ribosomal peptide synthetase [*Micrococcus luteus* (EFD52022)] (417, 56)	-	-
SA3	Amino acid adenylation domain-containing protein [*Micrococcus luteus* (CP001628)] (511, 62)	-	-
SA4	Amino acid adenylation domain-containing protein [*Micromonospora aurantiaca* (WP_013285021)] (501, 79)	Polyketide synthase [*Micromonospora* sp. CNB394 (WP_018787726)] (473, 67)	Type II PKS ketosynthase, partial [*Micromonospora* sp. SCSIO11524 (AHB18630)] (541, 63)
SA5	Amino acid adenylation domain-containing protein [*Salinispora arenicola* (WP_012184557)] (521, 72)	Polyketide synthase [*Salinispora arenicola* (WP_018795623)] (541, 64)	Beta-ACP synthase, partial [*Salinispora arenicola* (WP_020608853)] (573, 70)
SA6	Amino acid adenylation domain-containing protein [*Salinispora arenicola* (YP_001535283)] (513, 77)	Polyketide synthase [*Salinispora arenicola* (WP_018795623)] (516, 71)	KAS II [*Salinispora arenicola* (WP_020608853)] (581, 67)
* SA7	-	-	Polyketide synthase [*Nocardia nova* SH22a (AHH16328)] (614, 85)
* SA8	Non-ribosomal peptide synthetase [*Rhodococcus equi* (CBH48735)] (570, 69)	Putative polyketide synthase [*Rhodococcus equi* (WP_022593366)] (518, 65)	-
* SA9	Non-ribosomal peptide synthetase, partial [*Rhodococcus qingshengii* (WP_007730195)] (609, 68)	Putative polyketide synthase [*Rhodococcus opacus* B4 (BAH55256)] (428, 75)	-
SA10	-	-	-
* SA11	-	-	-
* SA12	Non-ribosomal peptide synthetase [*Rhodococcus opacus* (WP_005253470)] (622, 58)	Putative polyketide synthase [*Rhodococcus opacus* (BAH55256)] (428, 75)	-
* SA13	-	-	-
* SA14	Non-ribosomal peptide synthetase [*Kocuria rhizophila* (BAG29492)] (532, 74)	-	-
*SA15	Non-ribosomal peptide synthetase [*Kocuria rhizophila* (BAG29492)] (462, 68)	-	-
SA16	-	-	-
SA17	Non-ribosomal peptide synthetase [*Micrococcus luteus* (EFD52022) (423, 66)	-	-
SA18	-	-	-
SA19	Adenylation domain of nonribosomal peptide synthetase [*Salinispora pacifica* (WP_018724218)] (465, 58)	Polyketide synthase [*Salinispora pacifica* (WP_018824659)] (421, 59)	Beta-ACP synthase [*Salinispora pacifica* (WP_018720155)] (541, 61)
SA20	Amino acid adenylation domain-containing protein [*Salinispora arenicola* (WP_012184557)] (465, 58)	Polyketide synthase [*Salinispora arenicola* (WP_019032802)] (506, 61)	KAS II [*Salinispora arenicola* (WP_020608853)] (529, 57)
SA21	Nonribosomal peptide synthetase [*Saccharopolyspora spinosa* (WP_010314019)] (665, 74)	Polyketide synthase [*Saccharopolyspora spinosa* (WP_010311945)] (565, 69)	-
SA22	Amino acid adenylation domain of nonribosomal peptide synthetase [*Salinispora pacifica* (WP_018724218)] (476, 59)	Polyketide synthase [*Salinispora pacifica* (WP_018824659)] (501, 63)	Beta-ACP synthase [*Salinispora pacifica* (WP_018823591)] (531, 71)
SA23	Amino acid adenylation domain-containing protein [*Saccharopolyspora spinosa* (WP_010694383)] (643, 71)	Polyketide synthase [*Saccharopolyspora erythraea* (WP_011873765)] (533, 61)	-
SA24	Nonribosomal peptide synthetase [*Kocuria rhizophila* (WP_019309050)] (592, 73)	-	-
SA25	Amino acid adenylation domain-containing protein [*Salinispora arenicola* (YP_001539321)] (453, 68)	Rifamycin polyketide synthase [*Salinispora arenicola* (WP_020217874)] (578, 71)	Ketosynthase, partial [*Salinispora arenicola* AFO70123] (548, 63)

### 2.3. Anti-Infective Screening

Twenty-five actinomycete isolates were fermented in the medium, from which they were originally isolated, and ethyl acetate and methanol were used for extraction of secondary metabolites. The ethyl acetate and methanolic extracts were then screened against *Bacillus* sp. P25, *Escherichia coli* K12, *Fusarium* sp. P21, *Trypanosoma brucei* TC 221, *Leishmania major* and NS3 protease of West Nile Virus (Table 3). Nine actinobacterial extracts were active against at least one test pathogen. No activities were documented against *L. major*. Two isolates were active against *Bacillus* sp. and *E. coli* K12 while antifungal activities were reported for six extracts and anti-trypanosomal activity was documented for five extracts.

Two *Micrococcus* isolates were bioactive. Members of the genus *Micrococcus* were cultivated from diverse terrestrial and marine environments, however they are not well known for production of secondary metabolites. The antibiotic 2,4,4′-trichloro-2′-hydroxydiphenylether from sponge-associated *Micrococcus luteus* [48] and recently the thiazolyl peptide kocurin against methicillin-resistant *Staphylococcus aureus* [49]. Although the two *Micrococcus* isolates SA1 and SA3 are phylogenetically related (identical 16S rRNA sequences, Figure 2), their ethyl acetate extracts exhibited different biological profiles. This means that 16S rRNA gene as phylogenetic marker alone was not sufficient to distinguish genomic differences between actinomycete isolates sharing identical 16S rRNA gene sequence homologies and display different biosynthetic gene expression that could result in the production of different natural products [50].

The ethyl acetate extracts of *Salinispora* sp. SA6 and SA22 were active against almost all test pathogens, except *L. major*. The obligate marine *Salinispora* strains are prolific producers of structurally diverse natural products such as salinosporamide A from *S. tropica*, a potent proteasome inhibitor that has reached phase I clinical trials as an anticancer agent [51]. Other examples of bioactive compounds from various *Salinispora* species include arenimycin, rifamycins [52], cyanosporaside A, [53] saliniketals A and B [54], salinipyrones, and pacificanones [55]. The results highlight the high chemical potential of *Salinispora* isolates.

**Table 3:** Bioactivity results of the actinomycete isolates.

Isolate Code	Inhibition Zone Diameter (mm)			IC_50_ (μg/mL, 72 h)	Growth Inhibition (%)
	*Bacillus* sp. P25	*Escherichia coli* K12	*Fusarium* sp. P21	*Trypanosoma brucei* TC 221	West Nile Virus Protease
*Micrococcus* sp. SA1E	8	12	-	<10	-
*Micrococcus* sp. SA3E	-	-	14	-	-
*Salinispora* sp. SA6E	20	7	22	<10	84
*Salinispora* sp. SA22E	18	9	15	<10	79
* *Rhodococcus* sp. SA9E	-	-	13	-	-
* *Rhodococcus* sp. SA12E	-	-	16	-	93
*Mycobacterium* sp. SA11E	14	-	-	<10	-
*Saccharomonospora* sp. SA21 E	10	12	-	-	-
*Saccharomonospora* sp. SA23 E	11	13	17	<10	-

* Putatively new species.

Two extracts of the novel isolates *Rhodococcus* sp. SA9 and SA12 exhibited antifungal activity against *Fusarium* sp. P21 with *Rhodococcus* sp. SA12 showing additional activity against West Nile Virus NS3 protease. One isolate of the genus *Mycobacterium* showed activity against *Bacillus* sp. P25 as well as *Trypanosoma brucei* TC 221. The ethyl acetate extract of *Saccharomonospora* sp. SA21 was active against *Bacillus* sp. P25 and *Escherichia coli* K12 while *Saccharomonospora* sp. SA23 showed more broad activities against *Bacillus* sp. P25, *Escherichia coli* K12, *Fusarium* sp. P21, and *Trypanosoma brucei* TC 221.West Nile Virus (WNV) is a zoonotic virus which is widespread and endemic in Africa, the Middle East and western Asia as well as other parts of the world including United States, Europe and Australia [56]. There are commercially available animal vaccines, however up to date, no vaccines or treatments have been approved for human WNV infections [57]. This illustrates the urgent need to develop effective vaccines and antiviral drugs to prevent WNV infection in humans. The WNV protease NS3 is a prime target for antiviral drugs and has become the focus of considerable research efforts [57,58]. Interestingly, three ethyl acetate extracts (SA 6E, 22E, 12E) showed activity against WNV protease NS3 in the present study. Bioactivities were documented for ethyl acetate, but not methanolic extracts, which was consistent with literature reports showing that the majority of microbial natural products are secreted into the medium [59,60].

## 3. Experimental Section

### 3.1. Sponge Collection

Ten sponge species (*Amphimedon ochracea*, *Amphimedon* aff. *chloros*, *Acarnus wolffgangi*, *Chalinula* sp., *Crella cyathophora*, *Dactylospongia* aff. *elegans*, *Hyrtios erectus*, *Monanchora* sp., *Subera* sp., *Xestospongia* aff. *testudinaria*) were collected by SCUBA diving at depths of 8–12 m in the Red Sea (Saudi Arabia, Thuwal, Fsar Reef, GPS: 22°23′ N; 39°03′ E) in June 2012. Taxonomic identification was performed by Nicole de Voogd (Naturalis Biodiversity Center, Leiden, The Netherlands). Sponges were transferred to plastic bags containing sea water and transported to the laboratory. Sponge specimens were rinsed in sterile sea water, cut into pieces of *ca.* 1 cm^3^, and thoroughly homogenized in a sterile mortar with 10 volumes of sterile sea water. The supernatant was diluted in 10-fold series (10^−1^, 10^−2^, 10^−3^) and subsequently plated out on agar plates. Processing was equivalent among samples.

### 3.2. Actinomycete Isolation and Identification

M1, ISP medium 2 and Oligotrophic media (OLIGO) were used for actinomycete isolation as described previously [17]. All media were prepared in artificial sea water and were supplemented with cycloheximide (100 μg/mL) and nalidixic acid (25 μg/mL) to inhibit fungal growth and fast-growing Gram-negative bacteria, respectively. Actinomycetes were picked based on their morphological characteristics and re-streaked several times to obtain pure colonies. The isolates were maintained on plates for short-term storage and long-term strain collections were set up in medium supplemented with 30% glycerol at −80 °C. The isolates were abbreviated as “SA”.

DNA was extracted using the AllPrep DNA/RNA mini kit (QIAGEN, Hilden, Germany) following manufacturer’s instructions. 16S rRNA gene amplification and sequencing were performed using the universal primers 27F and 1492R. Chimeric sequences were identified using the Pintail program [61]. Raw sequences were processed on the software Sequencher 4.9 (Genecodes Coorperation, Ann Arbor, MI, USA). After ambiguous bases were trimmed to a quality over 99%, forward and reverse strands were assembled into a contig with the length of more than 1300 bases. Nearest related and described sequences were searched with an initial Blast run against the NCBI database. The genus-level identification of all the sequences was done with RDP Classifier (-g 16srrna, -f allrank) [62] and validated with the SILVA Incremental Aligner (SINA) (search and classify option) [63]. An alignment was calculated again using the SINA web aligner (variability profile: bacteria). Gap-only position were removed with trimAL (-noallgaps) [64]. For phylogenetic tree construction, the best fitting model was estimated initially with Model Generator [65]. RAxML (-f a -m GTRGAMMA –x 12345 –p 12345 -# 1000) [66] and the estimated model was used with 1000 bootstrap resamples to generate the maximum-likelihood tree. Visualization was done with TreeGraph2 [67]. The 16S rRNA gene sequences of the putatively novel isolates were deposited in GenBank under the accession numbers showed in parentheses: SA7 (KJ599861), SA8 (KJ599862), SA9 (KJ599863), SA11 (KJ599864), SA12 (KJ599865), SA13 (KJ599866), SA14 (KJ599867), and SA15 (KJ599868).

### 3.3. PCR Screening of NRPS and PKS-II Gene Fragments

Ketosynthase (KS) domains of type I polyketide synthase (PKS) gene were PCR amplified from genomic DNA using the primers K1F (5′-TSAAGTCSAACATCGGBCA-3′) and M6R (5′-CGCAGGTTSCSGTACCAGTA-3′). Type II PKS sequences were amplified using KSαF (5′-TSGRCTACRTCAACGCSCACGG-3′) and KSβR (5′-TACSAGTCSWTCGCCTGGTTC-3′). In order to target adenylation domains of NRPS genes, the degenerate PCR primers A3F (5′-GCSTACSYSATSTACACSTCSGG-3′) and A7R (5′-SASGTCVCCSGTSCGGTAS-3′) were used [68]. Sequences of the corresponding PCR products (KS domains, 1250–1400 bp; KSα and KSβ, 800–900 bp; adenylation domains, 700 bp) [69] were compared with NRPS and PKS sequences in the NCBI database by using the Basic Local Alignment Search Tool X (BLASTX).

### 3.4. Secondary Metabolites Extraction and Bioactivity Testing

Twenty-five strains were cultured in 500 mL Erlenmeyer flasks containing 250 mL of the appropriate cultivation medium for each isolate. The liquid cultures were grown for 7–10 days depending on their growth rate at 30 °C while shaking at 150 rpm. After cultivation and filtration, the supernatant was extracted with ethyl acetate (2 × 150 mL). The cells were macerated in 100 mL methanol and shaken for 3 h (150 rpm) then filtered. The extracts (ethyl acetate and methanol) were concentrated under vacuum and stored at 4 °C.

#### 3.4.1. Antibacterial and Antifungal Activities Testing

Antimicrobial activity was tested using the standard disk diffusion assay against *Bacillus* sp. P25, *Escherichia coli* K12 and *Fusarium* sp. P21. Sterile filter disks (6 mm) loaded 3 times with the test extracts (25 μL, 20 mg/mL in methanol) were placed on agar plates that had been inoculated with the test microorganism. After incubation (24 h for *Bacillus*, *Escherichia coli* K12 and 48 h for *Fusarium* sp.) at 37 °C (*Bacillus*, *Escherichia coli* K12) and 30 °C (*Fusarium* sp.), the antimicrobial potential was quantitatively assessed as diameter of the inhibition zone (*n* = 2).

#### 3.4.2. Anti-Trypanosomal Activity

Anti-trypanosomal activity was tested following the protocol of Huber and Koella [70]. 10^4^ trypanosomes per mL of *Trypanosoma brucei brucei* strain TC 221 were cultivated in Complete Baltz Medium. Trypanosomes were tested in 96-well plate chambers against different concentrations of test extracts at 10–200 μg/mL in 1% DMSO to a final volume of 200 μL. For controls, 1% DMSO as well as parasites without any test extracts were used simultaneously in each plate to show no effect of 1% DMSO. The plates were then incubated at 37 °C in an atmosphere of 5% CO_2_ for 24 h. After addition of 20 μL of Alamar Blue, the activity was measured after 48 and 72 h by light absorption using an MR 700 Microplate Reader (Dynatech Engineering Ltd., Willenhall, UK) at a wavelength of 550 nm with a reference wavelength of 650 nm. The IC_50_ values of the test extracts were quantified by linear interpolation of three independent measurements.

#### 3.4.3. Anti-Leishmanial Activity

Anti-leishmanial activity was tested following the method of Ponte-Sucre *et al.* [71]. 10^7^ cells/mL *Leishmania major* promastigotes were incubated in complete medium for 24 h at 26 °C, 5% CO_2_, and 95% humidity in the absence or presence of different concentrations of the test extracts (10–200 μg/mL, 1% DMSO) to a final volume of 200 μL. Following the addition of Alamar Blue, the plates were incubated again and the optical densities were determined after 48 h with a Multiskan Ascent enzyme-linked immunosorbent assay (ELISA) reader (Multiskan Ascent, Thermo Electron Corporation, Dreieich, Germany). The effects of cell density, incubation time and the concentration of DMSO were examined in control experiments. The results were expressed in IC_50_ values by linear interpolation of three independent experiments.

#### 3.4.4. West Nile Virus NS3 Protease Inhibition Assay

The West Nile Virus NS3 protease inhibition assay was carried out using the commercial kit SensoLyte^®^ 440 West Nile Virus Protease Assay Kit (AnaSep, San Jose, CA, USA) [58]. The quantification of protease activity was measured by using the fluorogenic peptide Pyr-RTKR-AMC which produces free AMC (7-amino-4-methylcoumarin) fluorophore upon NS3 protease cleavage. The extracts and protease solution were applied to 384-well plates and the total reaction mixture in each well was 40 μL. All extracts and controls were performed with three replicates and were generated according to the manufacturer’s instructions. Briefly, the test extracts and protease solution were mixed and incubated at 37 °C for 10 min before adding the fluorogenic substrate. After substrate addition, the reagents were completely mixed and incubated at 37 °C for one hour. The fluorescence intensities were measured using a SpectraMax^®^ Paradigm^®^ Multi-mode Microplate Detection Platform (Molecular Devices, Sunnyvale, CA, USA) at 354 nm (excitation) and 442 nm (emission).

## 4. Conclusions

Marine actinomycetes, such as those associated with marine sponges, are a rich source of bioactive natural products. In the present study, we isolated 47 actinomycetes representing 10 different genera and including eight putatively novel phylotypes. The isolates were obtained from sponges which were collected offshore Fsar reef, Saudi Arabia, a largely uncharted territory with respect to bioprospecting. Although 80% of actinomycetes contained at least one class of NRPS or PKS gene, antimicrobial activity was detected only for 36% of the isolates. This suggests that genomic mining is a worthwhile future endeavour. Bioactivities against bacteria, fungi, human parasites as well as West Nile Virus protease were reported for nine of the isolates. These results underscore the potential of Red Sea sponges as a source of novel actinomycetes with underexplored potential for drug discovery. The combination of PCR-based screening, phylogenetic analysis as well as bioactivity assays is a useful strategy to prioritize actinomycetes for further bioassay-guided isolation work.
